# Physicians’ attitudes towards office-based delivery of methadone maintenance therapy: results from a cross-sectional survey of Nova Scotia primary-care physicians

**DOI:** 10.1186/1477-7517-9-20

**Published:** 2012-06-13

**Authors:** Jessica Dooley, Mark Asbridge, John Fraser, Susan Kirkland

**Affiliations:** 1 Department of Community Health and Epidemiology, Faculty of Medicine, Dalhousie University, Halifax, NS B3H 1V7, Canada; 2 North End Community Health Centre, Halifax, NS B3K 3B5, Canada; 3 Department of Medicine, Faculty of Medicine, Dalhousie University, 5790 University Ave, Halifax, NS B3H 1V7, Canada

**Keywords:** Methadone Maintenance Therapy (MMT), Primary care physicians, Office-based delivery, Access, Barriers

## Abstract

**Background:**

Approximately 90,000 Canadians use opioids each year, many of whom experience health and social problems that affect the individual user, families, communities and the health care system. For those who wish to reduce or stop their opioid use, methadone maintenance therapy (MMT) is effective and supporting evidence is well-documented. However, access and availability to MMT is often inconsistent, with greater inequity outside of urban settings. Involving community based primary-care physicians in the delivery of MMT could serve to expand capacity and accessibility of MMT programs. Little is known, however, about the extent to which MMT, particularly office-based delivery, is acceptable to physicians. The aim of this study is to survey physicians about their attitudes towards MMT, particularly office-based delivery, and the perceived barriers and facilitators to MMT delivery.

**Methods:**

In May 2008, facilitated by the College of Physicians and Surgeons of Nova Scotia, a cross-sectional, e-mail survey of 950 primary-care physicians practicing in Nova Scotia, Canada was administered via the OPINIO on-line survey software, to assess the acceptability of office-based MMT. Logistic regressions, adjusted for physician sociodemographic characteristics, were used to examine the association between physicians’ willingness to participate in office-based MMT, and a series of measures capturing physician attitudes and knowledge about treatment approaches, opioid use, and methadone, as well as perceived barriers to MMT.

**Results:**

Overall, 19.8% of primary-care physicians responded to the survey, with 56% who indicated that they would be willing to be involved in MMT under current or similar circumstances; however, willingness was associated with numerous attitudinal and systemic factors. The barriers to involvement in MMT that were frequently cited included a lack of training or experience in MMT, lack of support services, and potential challenges of working with an MMT patient population.

**Conclusions:**

Study findings provide valuable information to help facilitate greater involvement of primary-care physicians in MMT, while highlighting concerns around administration, support, and training. Even limited uptake by primary-care physicians would greatly enhance MMT access in Nova Scotia, particularly for methadone clients located in rural communities. These findings are applicable broadly, to any jurisdictions where office-based MMT is not currently available.

## Background

The impact of opioid use on Canadians is substantial; the economic costs are high and health risks include transmission of blood borne disease, overdose, premature mortality and a host of other conditions [1-5]. Methadone, a long-acting synthetic opioid agonist, is an effective treatment for opioid dependence, and evidence of positive health outcomes for opioid users and their surrounding communities is well-documented [6-13]. However, methadone maintenance therapy (MMT) remains controversial and underutilized, and the degree to which MMT policies and programs are available, accessible, and adopted varies significantly between geographical and professional settings and facilities across the country.

A recent spate of prescription opioid drug-related deaths in Nova Scotia has received attention from the Minister of Health and a promise for more MMT spots [14]. Data on 431 drug-related deaths in Nova Scotia from Jan 2009 to Dec 2011 points to the heavy involvement of prescription opioids. Hydromorphone contributed to 19% of drug-related deaths, morphine 11%, and oxycondone 9%, compared with only one heroin-related death (C. Davidson, personal communication, May 22, 2012). Data on opioid treatment show a similar pattern. Treatment data from 2009–2010 indicate that 1272 Nova Scotians received treatment for opiate dependence, fewer than 10 of which involved heroin [15]. These numbers represent a substantial increase from just 10 years earlier [1].

Few primary-care physicians (general practitioners and family physicians) in Nova Scotia offer MMT and the capacity of community-based programs is limited, particularly outside of larger urban settings. As a result, many opioid-dependent persons who could benefit from MMT go untreated. Office-based MMT delivery has been suggested as an alternative to traditional specialized outpatient clinics that may aid in improving accessibility, reducing stigma and addressing the diverse needs of the opioid-using population [16-22]. Clinic-based delivery limits access to users who lack adequate transportation or for those who live a great distance from the clinic, disrupting normal daily routines as users must spend a great deal of time and resources just to obtain MMT. Office-based MMT delivery through a primary-care physician alleviates many of these issues, particularly if physicians are spread optimally around urban and rural settings in a given region.

Methadone maintenance is the main pharmacotherapeutic treatment for opioid dependence offered in Canada, although buprenorphine and other therapeutics treatments are available [23]. The delivery of methadone in Canada, including physician authorization, certification and MMT training, is regulated at the provincial level. As such, there is tremendous variability in availability and access across provinces. While the integration of MMT into primary care settings is encouraged, most MMT is still delivered through specialized clinics [24]. Office-based methadone maintenance delivered by primary-care physicians has been implemented in parts of Canada [20,25,26], and evidence points to its feasibility and effectiveness [11,21,27], including its cost effectiveness [28]. Treatment may be integrated into a dynamic community-based model of care [20,29] or patients, who have been stabilized on methadone, may be referred to a primary-care physician for continued but less intensive care [19,29].

The success of such a model is dependent in large part on the willingness of primary care physicians to deliver MMT as part of their office-based practice. As identified in Health Canada’s [24] “Best Practices” document for MMT, *program acceptability* is essential to improving MMT access and delivery. While office-based MMT delivery has expanded in Ontario and British Columbia, acceptability and, in turn, access in other parts of Canada remains limited. This study seeks to clarify the acceptability of MMT delivery among primary-care physicians in Nova Scotia, to determine their willingness to participate in office-based MMT, and to identify the barriers to participation in MMT delivery. The degree to which MMT practice is espoused by physicians sheds light on the potential for office-based MMT as a potential solution to access, while the perceived barriers to office-based delivery of MMT identifies unmet needs and social and political barriers. We address here the factors that may influence effective implementation of MMT, and highlight areas for improved treatment accessibility.

## Methods

### Study design and participants

We conducted a cross-sectional survey of primary-care physicians licensed in Nova Scotia, the objective of which was to assess acceptability of office-based MMT. The survey, developed in conjunction with the Nova Scotia College of Physicians and Surgeons, and administered by the College via e-mail in May 2008, was sent to 950 primary-care physicians including family physicians, general practitioners, and emergency room physicians. The names and addresses of the individual physicians were not provided to the research team, and all responses were anonymous.

Efforts were made to keep the survey as concise and efficient as possible [30]. The survey asked 29 questions on a range of topics around MMT knowledge, attitudes towards drug use, and perceived barriers, and took, on average, 20 minutes to complete. Content validity of the survey was assessed by creating a matrix that represented the domains to be measured and ensuring that each domain had adequate coverage [31]. In order to validate the scale for use in the Canadian context, experts in the field of methadone maintenance were asked to assess face and content validity for questions regarding barriers and facilitators to MMT delivery. Fifteen individuals, purposively sampled from the study population, participated in a pre-test of the survey; minor changes were made based on their feedback.

Approval for this study was obtained from the Office of Human Research Ethics at Dalhousie University, and from the Nova Scotia College of Physicians and Surgeons.

### Data collection

OPINIO on-line survey software (Version 6.4.4, ObjectPlanet, Inc: Oslo, Norway) was used to administer the survey. A link to the survey was e-mailed by the College of Physicians and Surgeons of Nova Scotia to all primary-care physicians with an e-mail address on record. The survey was preceded by a letter of explanation that also served as a letter of consent and highlighted the potential of the results to which participants would be contributing. Individuals were e-mailed first on Thursday May 15th, 2008; a follow-up reminder was sent ten days later.

A total of 189 responses were received, 130 of which were complete. This resulted in a response rate of 19.8% overall and 13.7% for complete responses. However, it is important to note that the true denominator with which to calculate the response may be lower as the e-mail list may include individuals that do not qualify to complete the survey (e.g. retired physicians still registered with the College). Nonetheless, the low response rate likely introduces response bias to the survey, as physicians with stronger opinions, knowledge, and experiences with MMT are more likely to have responded. Previous surveys conducted by the College of Physicians and Surgeons of Nova Scotia have had a response rate between 17 and 30% (personal communication).

Physicians over 50 years of age represented the largest proportion of respondents (35%), followed by those 41–50 (31%), 31–40 (23%), and those under 30 (11%). Just over half of participants were male (54%). Almost half of the respondents practiced in communities of over 50,000 population (Halifax and Sydney), while 40% practiced in communities of 5,000-50,000 people, and 13% practiced in communities of under 5,000 people. Practice setting varied with 20% involved in a solo private practice, 38% in a group private practice, and 41% in some other type of practice.

Only 14 participants (7%) had ever held a license to provide methadone for opioid dependence. The majority of participants (almost three quarters) had minimal training in addiction medicine or methadone maintenance, primarily workshops or lectures on addiction medicine and/or self-study. A small proportion had attended a course in addiction medicine and/or methadone maintenance.

### Outcome measures

The primary outcome of interest was ‘acceptability of office-based MMT’, as measured by willingness to participate in office-based MMT. Participants were asked to indicate one statement that most accurately reflected their current willingness to prescribe methadone and manage opioid dependent patients in their office-based practice. The statements were: “I would potentially be willing to provide office-based methadone maintenance treatment under current circumstances”; “I would potentially be willing to provide office-based methadone maintenance treatment under different circumstances”; and “I would not be willing to provide office-based methadone maintenance treatment under any circumstances”. Responses were dichotomized into those willing (either current or different circumstance) to provide MMT and those unwilling.

### Explanatory measures

Acceptability of office-based MMT was measured in relation to a number of independent variables thought to be associated with willingness. These included: a) attitudes towards treatment philosophy (commitment to abstinence-oriented approaches to treatment versus maintenance-oriented approaches), b) attitudes towards opioid users and opioid dependence, c) knowledge of the risks and benefits of methadone maintenance treatment (including office-based MMT) and d) perceived barriers to office-based MMT.

Attitudes towards treatment were measured with the Abstinence Orientation scale (see Table 1, Column 1), which consists of 14 items that reflect two dimensions of support for abstinence oriented maintenance; an ‘abstinence goal’ measuring a commitment to the goal of abstinence and ‘compliance’, measuring support for the use of disciplinary sanctions to enforce compliance with program rules [32]. The Disapproval of Drug Use scale (Table 1, Column 2) consists of six items that are concerned with attitudes towards punitive treatment of illicit drug use. All items are attached to a Likert scale responses are scaled between 1 (strongly agree) and 5 (strongly disagree). The final score is reflective of the summed score for all items divided by the total number of items (14 and 6, respectively), thus the range of final scores for each item is between 1 and 5. These scales were developed and validated in Australia, though neither has been used previously in Canada. However, tests of the internal reliability of scale items (Abstinence Orientation scale α = 0.74; Disapproval of Drug Use scale α = 0.63) were moderate to strong [30].

**Table 1:** Items in the Abstinence Orientation, Disapproval of Drug Use, and Knowledge of MMT scales

**Abstinence Orientation Scale**	**Disapproval of Drug Use Scale**	**Knowledge of the risks and benefits of MMT**
1) Methadone maintenance patients who continue to use illicit opiates should have their dose of methadone reduced.	1) Marijuana should be legalized.	1) Methadone, in a stable dose as partof a maintenance regime, blocks the euphoric effects of heroin and prescription opioids.
2) Maintenance patients who ignore repeated warning to stop using illicit opiates should be gradually withdrawn off methadone.	2) Modern society is too tolerant toward drug addicts.	2) Withdrawing from methadone ‘cold turkey’ is definitely worse than withdrawing from heroin.
3) No limits should be set on the duration of methadone maintenance.	3) Drug addiction is a vice.	3) Methadone maintenance can cause chronic constipation.
4) Methadone should be gradually withdrawn once a maintenance patient has ceased using illicit opiates.	4) Marijuana use among teenagers can be healthy experimentation.	4) Methadone Maintenance can cause disturbance of sexual function.
5) Methadone services should be expanded so that all narcotic addicts who want methadone maintenance can receive it.	5) Drug addiction is a menace to society.	5) Methadone maintenance can cause kidney damage.
6) Methadone maintenance patients whocontinue to abuse non-opioid drugs (e.g. benzodiazepines) should have their dose of methadone reduced.	6) Persons convicted of the sale of illicit drugs should not be eligible for parole.	6) Methadone maintenance can cause liver damage.
7) Abstinence from all opioids (including methadone) should be the principal goal of methadone maintenance.		7) To the unborn child, methadone is more dangerous than heroin.
8) Left to themselves, most methadone patients would stay on methadone for life.		8) Methadone given in a stable dose aspart of a maintenance regime significantly interferes with the ability to dive a car.
9) Maintenance patients should only be given enough methadone to prevent the onset of withdrawals.		9) Methadone maintenance reduces addicts’ criminal activities.
10) It is unethical to maintain addicts on methadone indefinitely.		10) Methadone maintenance decreases addicts’ risk of dying.
11) The clinician’s principal role is to prepare methadone maintenance patients for drug-free living.		11) Methadone maintenance reduces addicts’ consumption of illicit opiates.
12) It is unethical to deny a narcotic addict methadone maintenance.		12) Methadone maintenance increases the severity of preexisting depression.
13) Confrontation is necessary in the treatment of drug addicts.		13) Methadone maintenance reduces the risk of transmission blood borne diseases.
14) The clinician should encourage patients to remain in methadone maintenance for at least three to four years.		

Knowledge of the risks and benefits of MMT were measured with a Knowledge scale (Table 1, Column 3) consisting of 12 statements regarding MMT. The original scale contained 13 items, however one question was deemed to be ambiguous. Responses to each statement were either true, false, or uncertain, with one point awarded for correct answers, one point subtracted for incorrect answers, and no points were given for uncertain responses [33], and thus scores on the knowledge could range from −12 to +12.

Finally, perceived barriers to office-based MMT were assessed using the Barriers to Methadone Prescribing scale, a tool developed for this study. The Barriers to Methadone Prescribing scale was comprised of 16 questions drawn from the purposively sampled group of physicians and previous research on barriers to MMT among physicians [26,34-40]. These questions tapped into a range of common barriers to treatment that have been previously identified, such as belief in the effectiveness of methadone, availability of support from specialists, availability of resources, and concerns about this patient population (See Table 1). As above, items were measured on a Likert scale (strong agree to strongly disagree), where each item was given a score between 1 and 5.

Given that this was a previously unvalidated scale, and due to the high correlation between items, a principal components analysis was performed. Rather than eliminating some of the variables from the analysis, and thus reducing the amount of variance that can be explained, principal components analysis made it possible to create principal component scores, which are uncorrelated linear combinations of weighted observed variables, and therefore explain as much variance as possible. The principal component analysis found that the 16 items loaded onto three distinct factors (see Table 2 for factors/loadings), representing concerns about: 1) risks associated with MMT delivery (α = 0.80); 2) training and support issues (α = 0.75); and 3) resistance from external sources (α = 0.82).

**Table 2:** Principal components analysis of items in Barriers to Methadone Prescribing Scale

**Factor names and items**	**Factor Loadings**		
**Risks associated with MMT delivery**	**Factor 1**	**Factor 2**	**Factor 3**
1. Don’t want it known that I have a methadone license	0.74*	0.27	0.19
2. It would shift my practice too much to patients with opioid dependence	0.81*	0.13	0.02
3. Afraid of patient diversion of methadone	0.75*	0	−0.05
4. Fear of becoming involved with surveillance	0.58*	0.13	0.47
5. Am generally uncomfortable managing patients with opioid dependence	0.55*	0.01	0.33
6. Difficult patient population	0.57*	0.17	0.15
**Training and support issues**			
7. Not enough reimbursement	0.13	0.46*	0.32
8. Not enough interaction with other methadone maintenance practitioners	−0.02	0.54*	0.23
9. Not enough political commitment	0.15	0.54*	0.31
10. Not enough support services (e.g. drug screening, addiction counseling services)	0.03	0.62*	0.19
11. Too much paperwork	0.27	0.80*	0.04
Too much time involved	0.37	0.76*	−0.13
Little or no experience, training or education in the use of methadone for opioid dependence	0.01	0.51*	−0.10
**Resistance from external sources**			
Community resistance	0.13	0.03	0.90*
Staff resistance	0.17	0.05	0.86*
Extensive regulations	0.14	0.34	0.71*

*Loading used for interpretation of factors.

Additionally, we included a number of variables reflective of physician’s sociodemographic (age, gender) profile, location of training (North America or other), practice setting (solo, group private, clinic, other), size of the community in which they practice (under 5,000, 5,000 to 50,000, over 50,000 people), whether they received formal training in addiction medicine, and whether they are licensed to prescribe methadone. Open-end questions were also included in each section of the survey to allow physicians to offer additional responses not captured in the close-ended questions or to offer insight and opinions on the subject matter.

### Statistical analysis

Analysis followed two stages. First, descriptive statistics were used to summarize our outcome and main explanatory variables. Second, the association between willingness to prescribe methadone (measured dichotomously) and knowledge of the risks and benefits of MMT, attitudes to illicit drug use (DDU), attitudes to MMT (AO), and the three factors in the perceived barriers to MMT delivery, was examined with logistic regression. Unadjusted associations with other covariates (age, gender, location of training, community size, addictions medicine training) were also obtained. Employing a likelihood ratio test, and a *p* value threshold of 0.25, covariates that did not contribute to the model were eliminated from a final adjusted logistic model. Key explanatory measures were retained in all models. Logistic models present odds ratios and 95% confidence intervals, and include only those 130 physicians who answered all questions in the survey. All data analysis was performed using SAS statistical software (SAS Institute Inc., Cary, NC, USA. 2008, Version 9.1).

## Results

Overall, sixty-eight individuals (57%) were ‘willing’ to prescribe methadone and 51 (43%), were ‘unwilling’. In terms of explanatory measures, both the mean score on the Disapproval of Drug Use (DDU) scale (mean 3.25, SD 0.69) and the Abstinence Orientation (AO) scale (mean 3.00, SD 0.54) score were above 3 (scored out of a possible 5 points), with higher scores indicating a tendency towards disapproval of illicit drug use and an orientation towards abstinence. The mean score on the Knowledge scale was low (6.25 out of a possible 12 points, SD 3.50), demonstrating that additional training in the risks and benefits of methadone maintenance would likely be beneficial.

The correlation between the AO scale and the DDU scale was 0.51 (*p* = <0.0001) indicating that a higher score on the AO scale is associated with a higher score on the DDU scale. A higher score on the Knowledge scale was associated with lower scores on the AO and DDU scales. The correlation between the AO scale score and the Knowledge scale score was −0.34 (*p* = <0.0001) while the correlation between the DDU scale score and the Knowledge scale was −0.186 (*p* = 0.025).

As described above, while principal components analysis of items in the Barriers to Methadone Prescribing scale revealed three distinct factors, it is important to note specific results for some of the items in the scale. Perceived barriers for which there was a high level of agreement included perceptions of a ‘lack of support services’ (66% agreement), having ‘little or no experience’ with the population (69% agreement), and the belief that methadone users were a ‘difficult patient population’ (69%). Primary-care physicians disagreed with the perception that methadone is not effective (66%), and that they had a ‘fear of surveillance’ (40%), or that there was ‘community resistance’ (44%) and ‘resistance from other staff’ (44% overall). The mean score for the factors was calculated by adding the individual item scores, dividing by the number of items included in that factor, and by the number of responses. This resulted in a standardized score out of 5 for each factor, a higher score signifying higher level of agreement. The mean score for ‘training and support’ was highest, with a mean score of 3.37. The mean score for ‘risks’ was 3.22, and the mean score for ‘external resistance’ was lowest at 2.80.

Unadjusted results are summarized in Table 3. It was found that being female (OR = 3.04, 95% C.I. 1.41-6.56) and ‘training and support’ on the Barriers to Methadone Prescribing scale (OR = 1.59, 95% C.I. 1.04–2.43) were significantly positively associated with willingness to prescribe methadone. Conversely, a significant negative association was observed between willingness to prescribe, the DDU scale (OR 0.61, 95% C.I. 0.37–0.99), and the AO scale (OR 0.59, 95% C.I. 0.38–0.93). The direction of the association indicates that greater agreement with these items is associated a lower willingness to prescribe methadone. All remaining explanatory measures were not significantly associated with willingness to prescribe methadone.

**Table 3:** Logistic regression of willingness to prescribe methadone on scales and other covariates (n = 119)

	**Unadjusted**			**Adjusted**		
**Explanatory Measures**	**OR**	**95% CI**		**OR**	**95% CI**	
Age (referent = over 51)						
Under 30	4.29	0.82	22.31			
31–40	1.13	0.41	3.09			
41–50	1.32	0.56	3.13			
Sex (referent = male)						
Female	3.04	1.41	6.56	3.75	1.55	9.01
Community Size (referent = over 50,000)						
Under 5,000	0.66	0.22	2.00			
5,000–50,000	0.53	0.24	1.18			
Location of Graduation (referent = Other)						
North America	2.52	0.85	7.47			
Type of Practice (referent = Other)						
Group or solo private practice	0.83	0.40	1.75			
Knowledge Test score	1.09	0.73	1.61	1.11	0.69	1.78
Education in Addiction Medicine	1.86	0.88	3.93			
Disapproval of Drug Use Scale factor score	0.61	0.37	0.99	0.60	0.32	1.13
Abstinence Orientation Scale factor score	0.59	0.38	0.93	0.83	0.46	1.48
Barriers to Methadone Prescribing Scale						
Risks associated with MMT deliveryfactor	0.69	0.46	1.03	0.77	0.49	1.22
Training and support issues factor	1.59	1.04	2.43	1.63	1.04	2.54
Resistance from external sources factor	0.98	0.65	1.46	0.95	0.61	1.47

An adjusted model found being female (OR = 3.75, 95% C.I. 1.55–9.01) and the ‘training and support’ factor of the Barriers to Methadone Prescribing scale (OR = 1.63, 95% C.I. 1.04–2.54) were the only explanatory measures significantly associated with willingness to prescribe methadone.

Physicians were also given the opportunity to provide open-ended comments. While some physicians expressed comfort and skill with working with the population, others expressed frustration at the current state of MMT delivery. For example one physician stated “In general I strongly support changes to the existing regulations to make methadone more easily and widely available. The existing paranoid treatment of methadone as a ‘special’ drug, requiring specific licensing, has no basis in science or rationality.” This sentiment was echoed by another physician: “Once the state legalizes currently illegal drugs, and gets out of the way of decent physicians trying to help their patients, then I would be willing to offer a methadone treatment service, and not before. Getting on board now simply aids and abets the status quo”.

Issues of administrative support and training were also reflected upon in responses to open-ended questions in the survey. One physician working in the context of Correctional Services commented on the lack of sufficient community resources to sustain individuals once they have returned to their communities and noted that women especially have a problem finding services near their families or halfway houses in communities in which methadone services are available. Some physicians noted that MMT is a politically charged issue, expressing concern about the degree of support they would receive should complaints be made. Others cited concern about systemic support and remuneration. Others spoke of broader prevention and improved population health, noting the need for “upstream” investment in “strategies that support families and community, to improve early childhood development, deal with learning disabilities, etc., and so work to prevent the difficulties that lead to involvement with addiction”.

## Discussion

We found a considerable level of willingness (56%) to participate in methadone maintenance treatment among a sample 189 (response rate of 19.8%) primary-care physicians in Nova Scotia. Of these physicians, only 15% indicated that they would be willing to participate in MMT under current circumstances, while 44% indicated that they would be unwilling to participate in MMT under any circumstances. To contextualize findings further we employed a series of scales to examine primary-care physicians’ attitudes towards illicit drug use and abstinence orientation, and their knowledge of MMT. Overall, the mean Disapproval of Drug Use scale score indicated considerable disapproval of illicit drug use among primary-care physicians, and those physicians with higher disapproval scores were significantly less willing to prescribe methadone. Similarly, a high mean score was found on the Abstinence Orientation scale, demonstrating that primary-care physicians prefer abstinence-based treatment for substance users rather than maintenance (harm reduction) approaches. A high abstinence orientation was also negatively associated with a willingness to prescribe methadone. We found, overall, that primary-care physicians had limited knowledge of methadone maintenance therapy; however knowledge was not associated with willingness to prescribe methadone. We identified numerous barriers to office-based methadone maintenance treatment, and of particular significance were issues related to training and support.

Considered along with low knowledge levels, a higher orientation to abstinence-based therapies may indicate, among other things, that best practices in MMT are poorly disseminated and not reaching physicians, or reflective of limited addictions training in medical education. Of course, it may simply be that physicians’ less than positive attitudes and values about MMT and illicit opioid use holds influence on their opinions about clinical decisions [41]. Enhancements to both undergraduate medical training and continuing medical education may represent reasonable approaches to shaping clinical practice in this area, particularly since there is a willingness to become educated: forty percent of primary-care physicians surveyed indicated that they would be willing to be trained in MMT, preferably by academic detailing, group seminars, regular symposia and time spent with a mentor. A study from the United Kingdom found that the publication of clear and concise best practice guidelines for methadone and buprenorphine prescribing coincided with a doubling of methadone prescriptions and considerable improvements on a number of clinical decision-making outcome measures [42].

Potential barriers to primary-care physician participation in the delivery of office-based MMT were also examined. The barriers most frequently identified in the survey addressed a lack of experience or training, the difficult nature of the patient population, a lack of support services, and the need for more interaction with other methadone providers, all of which have been previously identified [34-36]. In general, the administrative barriers highlighted in this study are systemic and are not specific to methadone prescribing, as highlighted by the 2007 National Physician Survey conducted by the College of Family Physicians of Canada [43].

Our study was cross-sectional and the response rate was limited, making it impossible to draw overarching conclusions about the willingness of primary-care physicians to participate in MMT at the population level. Non-response was analyzed by comparing the group of responders with the population of primary-care physicians in Nova Scotia, drawn from the Office of Continuing Medical Education (CME) at Dalhousie University, across a number of demographic characteristics. Results of this comparison indicate a comparable ratio of female to male physicians, though a strong divergence in age was observed. Survey respondents were considerably younger than the population of Nova Scotia primary-care physicians. Some of this discrepancy may be explained by the fact that current residents or recent graduates are not included in the list provided by CME, while they were included in the distribution of the survey. Likewise, retirees may not have received the survey or may have chosen not to complete the survey, but remain included in the CME list. Additionally, a larger proportion of survey respondents practiced in communities with a population 50,000 or more when compared to all primary-care physicians in Nova Scotia. This discrepancy may be explained by the perceived salience of this issue in communities of different sizes. Those from small communities may feel that opioid dependence is not a major issue in their communities and choose not to participate, while physicians from larger communities may perceive opioid dependence to be an issue of greater importance.

One additional limitation relates to the wording of questions on future prescribing intention. These questions excessively qualify the conditions under which a physician would be willing to prescribe MMT; as such, there is a potential uncertainty created by this question that may have biased results about physicians’ true intentions. Despite these concerns,, many of the study findings are valuable in that they offer insight and direction for medical education, and for the development of provincial strategies to respond to the limited access to MMT treatment in Nova Scotia and other jurisdictions. Overall, 68 physicians indicated their willingness to prescribe MMT under the right circumstances. If even half of these physicians could become active prescribers, access problems could be substantially reduced or eliminated.

## Conclusion

In conclusion, methadone maintenance treatment is a health service that can have a dramatic impact on individuals and their communities, but one that has traditionally not received full acceptance by the medical community. This is the image that was conveyed by primary-care physicians who responded to our survey. In general, our respondents’ attitudes leaned more towards disapproval of drug use and orientation to abstinence rather than maintenance therapy, both of which were associated with lower willingness to prescribe methadone. Overall, however, we found physicians willing to be educated and involved in MMT – though not necessarily given current political and administrative structures, poor resources, and lack of supports. Continuing Medical Education programs in MMT and clear, concise provincial guidelines on the administration of MMT may serve to support the provision of these services. The barriers highlighted by our respondents identify areas not only of systemic improvement needed, but also areas where more information and experience may serve to allay fears about risks associated with MMT provision. This must be aimed not only at current primary-care physicians, but also at the undergraduate medical education curriculum in order to improve awareness and understanding of addict populations and appropriate treatments.

We have also highlighted the potential of this mode of MMT delivery to improve MMT capacity and accessibility, such that primary-care physicians may be willing to be engaged in office-based MMT under appropriate conditions. Primary-care physicians have a role to play in the delivery of MMT but, based on our results, there are considerable perceived and real barriers standing in the way of realizing our full potential in implementing this much-needed service. The recent and tragic prescription opioid abuse-related deaths in Nova Scotia, and other areas, reinforce the salience of this issue and the timeliness of action.

## Competing interests

Jessica Dooley, Mark Asbridge, John Fraser, and Susan Kirkland declare that they have no competing interests.

## Authors’ contributions

JD conceived of the study, participated in the design and completion of the study, led the analysis of the results, and participated in the writing and preparation of the manuscript. MA participated in the design of the study and the analysis of the results, and was involved in the writing and preparation of the manuscript. JF participated in the design of the study and writing of the manuscript. SK conceived of the study, participated in the design and analysis of study results, and contributed to the writing and preparation of the manuscript. All authors read and approved the final manuscript.

## References

[B1] Popova S Rehm J Fischer B An overview of illegal opioid use and health services utilization in Canada Public Health 2006 120 320 328 10.1016/j.puhe.2005.09.010 16476455

[B2] Rehm J Baliuna D Brochu S Fischer B Gnam W Patra J Popova S Sarnocinska-Hart A Taylor B The costs of substance abuse in Canada 2002 2006 Canadian Centre on Substance Abuse, Ottawa, ON

[B3] Fischer B Brissette S Brochu S Bruneau J El-Guebaly N Noel L Rehm J Tyndall M Wild C Mun P Haydon E Baliunas D Determinants of overdose incidents among illicit opioid users in 5 Canadian cities Can Med Assoc J 2004 171 3 235 239 10.1503/cmaj.1031416 15289420 PMC490072

[B4] Hser Y Hoffman V Grella CE Anglin MD A 33-year follow-up of narcotic addicts Arch Gen Psychiatry 2001 58 503 508 10.1001/archpsyc.58.5.503 11343531

[B5] Millson P Challacombe L Villeneuve PJ Fischer B Strike CJ Myers T Shore R Hopkins S Raftis S Pearson M Self-perceived health among Canadian opiate users Can J Public Health 2004 95 2 99 104 15074898 10.1007/BF03405775 PMC6976227

[B6] Novick DM Richman BL Friedman JM Friedman JE Fried C Wilson JP Townley A Kreek MJ The medical status of methadone maintenance patients in treatment for 11–18 years Drug Alcohol Depend 1993 33 3 235 245 10.1016/0376-8716(93)90110-C 8261888

[B7] Kreek MJ Medical safety and side effects of methadone in tolerant individuals J Am Med Assoc 1973 223 6 665 668 10.1001/jama.1973.03220060039009 4739193

[B8] Strain EC Stitzer ML Liebson IA Bigelow GE Dose–response effects of methadone in the treatment of opioid dependence Ann Intern Med 1993 119 1 23 27 8498759 10.7326/0003-4819-119-1-199307010-00004

[B9] Thiede H Hagan H Murrill CS Methadone treatment and HIV and hepatitis B and C risk reduction among injectors in the Seattle area J Urban Health 2000 77 3 331 345 10.1007/BF02386744 10976608 PMC3456044

[B10] Dolan KA Shearer J MacDonald M Mattick RP Hall W Wodak AD A randomised controlled trial of methadone maintenance treatment versus wait list control in an Australian prison system Drug Alcohol Depend 2003 72 1 59 65 10.1016/S0376-8716(03)00187-X 14563543

[B11] Gossop M Marsden J Stewart D Kidd T The national treatment outcome research study (NTORS): 4–5 year follow-up results Addiction 2003 98 291 303 10.1046/j.1360-0443.2003.00296.x 12603229

[B12] Willner-Reid J Belendiuk KA Epstein DH Schmittner J Preston KL Hepatitis C and HIV risk behaviour in polydrug users on methadone maintenance J Subst Abuse Treat 2008 35 1 78 86 10.1016/j.jsat.2007.08.011 17931826 PMC2600879

[B13] Millson P Challacombe L Villeneuve PJ Strike CJ Fischer B Myers T Shore R Hopkins S Reduction in injection-related HIV risk after 6 months in a low-threshold methadone treatment program AIDS Educ Prev 2007 19 2 124 136 10.1521/aeap.2007.19.2.124 17411415

[B14] Canadian Press, May 13, 2011. “Nova Scotia government to expand methadone treatment after drug-related deaths.” http://www.ipolitics.ca/2011/05/13/nova-scotia-government-to-expand-methadone-treatment-after-drug-related-deaths/

[B15] Nova Scotia Addiction Services 2009–2010 Annual Report 2011 Nova Scotia Department of Health and Wellness, Halifax

[B16] Byrne A Wodak A Census of patients receiving methadone treatment in a general practice Addict Res 1996 3 4 341 349 10.3109/16066359609005247

[B17] Greenwood J Persuading general practitioners to prescribe - good husbandry or recipe for chaos? Br J Addict 1992 87 567 575 10.1111/j.1360-0443.1992.tb01958.x 1591510

[B18] Van Brussel G Methadone treatment in Amsterdam: The critical role of general practitioners Addict Res 1996 3 4 363 368 10.3109/16066359609005249

[B19] Novick DM Joseph H Salsitz EA Kalin MF Keefe JB Miller EL Richman BL Outcomes of treatment of socially rehabilitated methadone maintenance patients in physicians’ offices (medical maintenance): Follow-up at three and a half to nine and a fourth years J Gen Intern Med 1994 9 3 127 130 10.1007/BF02600025 8195910

[B20] Latowsky M Kallen E Mainstreaming methadone maintenance treatment: The role of the family physician Can Med Assoc J 1997 157 4 395 398 9275948 PMC1227912

[B21] Fiellin DA O’Connor PG Chawarski M Pakes JP Pantalon MV Schottenfeld RS Methadone maintenance in primary care: A randomized controlled trial JAMA 2001 286 14 1724 1731 10.1001/jama.286.14.1724 11594897

[B22] Salsitz EA Joseph H Frank B Perez J Richman BL Saloman N Kalin MF Novick DM Methadone medical maintenance (MMM): Treating chronic opioid dependence in private medical practice - A summary report (1983–1998) Mt Sinai J Med 2000 67 5&6 388 397 11064489

[B23] Fischer B Rehm J Patra J Crus MF Change in illicit opioid use across Canada CMAJ 2006 175 11 1385 1387 10.1503/cmaj.060729 17116905 PMC1635767

[B24] Best practices - methadone maintenance treatment. No. H49-164/2002E 2002 Health Canada, Ottawa, ON

[B25] Anderson JF Methadone maintenance: BC leads the way Can Med Assoc J 1997 157 9 1198 9361631 PMC1228336

[B26] Fischer B Cape D Daniel N Gliksman L Methadone treatment in Ontario after the 1996 regulation reforms Ann Med Intern 2002 153 7 2S11 2S21 12518078

[B27] Gossop M Marsden J Stewart D Lehmann P Strang J Methadone treatment practices and outcome for opiate addicts in drug clinics and in general practice results from the national treatment outcome research study Br J Gen Pract 1999 49 31 34 10622013 PMC1313314

[B28] Jones ES Moore BA Sindelarc JL O’Connora PG Schottenfeld RS Fiellin DA Cost analysis of clinic and office-based treatment of opioid dependence: Results with methadone and buprenorphine in clinically stable patients Drug Alcohol Depend 2009 99 132 140 10.1016/j.drugalcdep.2008.07.013 18804923 PMC2646001

[B29] van Brussel G Methadone treatment by general practitioners in Amsterdam Bull N Y Acad Med 1995 72 2 348 358 10101375 PMC2359435

[B30] Rossi PH Wright JD Anderson AB Handbook of survey research 1983 Academic, New York

[B31] Streiner DL Norman GR Health measurement scales: A practical guide to their development and use 1989 Oxford University Press, Oxford

[B32] Caplehorn JRM Irwig L Saunders JB Attitudes and beliefs of staff working in methadone maintenance clinics Subst Use Misuse 1996 31 4 437 452 10.3109/10826089609045820 8851811

[B33] Gjersing LR Butler T Caplehorn J Belcher JM Matthews R Attitudes and beliefs towards methadone maintenance treatment among Australian prison health staff Drug and Alcohol Review 2007 26 501 508 10.1080/09595230701499118 17701513

[B34] McKeown A Matheson C Bond C A qualitative study of GPs’ attitudes to drug misusers and drug misuse services in primary care Family Practice 2002 20 2 120 125 12651783 10.1093/fampra/20.2.120

[B35] McGillion J Wanigaratne S Feinmann C Godden T Byrne A GPs’ attitudes towards the treatment of drug misusers Br J Gen Pract 2000 50 385 386 10897536 PMC1313703

[B36] Pelet A Doll S Huissoud T Resplendino J Besson J Favrat B Methadone maintenance treatment in the Swiss Canton of Vaud: demographic and clinical data on 1,782 ambulatory patients Eur Addict Res 2005 11 99 106 10.1159/000083039 15785071

[B37] Hobden KL Cunningham JA Barriers to the dissemination of four harm reduction strategies: A survey of addiction treatment providers in Ontario Harm Reduct J 2006 3 35 1 20 10.1186/1477-7517-3-35 17169151 PMC1764412

[B38] Ogborne AC Birchmore-Timney C Support for harm-reduction among staff of specialized treatment services in Ontario, Canada Drug Alcohol Rev 1998 17 1 51 58 10.1080/09595239800187591 16203468

[B39] Ogborne AC Wild CT Braun K Newton-Taylor B Measuring treatment process beliefs among staff of specialized addiction treatment services J Subst Abuse Treat 1998 15 4 301 312 10.1016/S0740-5472(97)00196-7 9650138

[B40] Ruch JD Boutwell AE Shield DC Key RG McKenzie M Clarke JG Friedmann PD Attitudes and practices regarding the use of methadone in US and state federal prisons J Urban Health 2005 82 3 411 418 10.1093/jurban/jti072 15917502 PMC3456063

[B41] Weber EM Failure of physicians to prescribe pharmacotherapies for addiction: regulatory restrictions and physician resistance J Health Care L & Pol’y 2010 13 1 49

[B42] Strang J Manning V Mayet S Ridge G Best D Sheridan J Does prescribing for opiate addiction change after national guidelines? Methadone and buprenorphine prescribing to opiate addicts by general practitioners and hospital doctors in England, 1995–2005 Addiction 2007 102 5 761 770 10.1111/j.1360-0443.2007.01762.x 17506153

[B43] http://www.nationalphysiciansurvey.ca/nps/2007_Survey/2007nps-e.asp

